# Cisplatin-Induced Stria Vascularis Damage Is Associated with Inflammation and Fibrosis

**DOI:** 10.1155/2020/8851525

**Published:** 2020-09-22

**Authors:** Na Zhang, Jing Cai, Lei Xu, Haibo Wang, Wenwen Liu

**Affiliations:** Department of Otolaryngology-Head and Neck Surgery, Shandong Provincial ENT Hospital, Cheeloo College of Medicine, Shandong University, Jinan 250022, China

## Abstract

The stria vascularis (SV) generates the endocochlear potential (EP) in the inner ear and is necessary for proper hair cell (HC) mechanotransduction and hearing. Cell junctions are indispensable for the establishment of compositionally distinct fluid compartments in the inner ear. Ototoxic drug cisplatin can damage SV and cause sensorineural hearing loss; however, the underlying mechanisms behind such injury are unclear. In this study, after the intraperitoneal injection of cisplatin (3 mg/kg/day for 7 days) in mice, we determined the auditory function by EP recording and auditory brainstem response (ABR) analysis, observed the ultrastructure of SV by transmission electron microscopy (TEM), and examined the expression and distribution of cell junction proteins by western blot, PCR, and immunofluorescence staining. We discovered that the EP was significantly reduced while ABR thresholds were significantly elevated in cisplatin-treated mice; cisplatin induced ultrastructural changes in marginal cells (MCs), endothelial cells (ECs), pericytes, etc. We found that cisplatin insulted auditory function not only by reducing the expression of zonula occludens protein-1 (ZO-1) in MCs of the SV but also by decreasing the expression of connexin 26 (Cx26) and connexin 43 (Cx43) in MCs and basal cells (BCs). More importantly, cisplatin induced activations of perivascular-resident macrophage-like melanocytes (PVM/Ms) and interleukin-1beta (IL-1*β*) as well as increased expressions of profibrotic proteins such as laminin and collagen IV in SV. Thus, our results firstly showed that cisplatin induced fibrosis, inflammation, and the complex expression change of cell junctions in SV.

## 1. Introduction

The endolymph is the atypical potassium-rich extracellular fluid which results in a 80-millivolt positive potential known as the endocochlear potential (EP), an essential driving force for hearing function [1]. Stria vascularis (SV), a heterogenous and nonsensory epithelial tissue in the lateral wall of the cochlea, generates and maintains the high concentration of potassium in the endolymph. The intrastrial fluid-blood barrier separates the SV from peripheral circulation, and the integrity of the barrier is critical for maintaining inner ear homeostasis, especially for sustaining the EP [2]. Disruption of the barrier is closely associated with hearing disorders including autoimmune inner ear disease, noise-induced hearing loss, and age-related hearing loss [3, 4]. Studies have reported that particular cell types in SV were identified as critical to prevent the leakage of solutes and generate EP, thus far including marginal cells (MCs), intermediate cells (ICs), and basal cells (BCs) [5–7]. MCs face the endolymph and extend basolateral projections that interdigitate with ICs which have projections that run in both directions toward MCs apically and BCs at the basolateral end. BCs play a role in barrier formation by connecting to each other by gap junctions (GJs) to prevent leakage of ions. In addition, other cell types in the SV include perivascular-resident macrophage-like melanocytes (PVM/Ms), pericytes, and endothelial cells (ECs) [8, 9].

Cisplatin is a widely used chemotherapeutic drug effectively against a variety of tumor types. Unfortunately, cisplatin can cause serious ototoxic side effects by directly damaging the hair cells (HCs) and spiral ganglion neurons [10, 11], and it has been reported that cisplatin interfered with SV function, resulting in depression of the EP [12] and inhibition of membrane-bound enzyme systems [13]. Histologically, cisplatin-induced damages to the SV consist of PVM/M atrophy, swelling and blebbing of the MCs, and vacuolation of the latter's cytoplasm [14]. Alam et al. have revealed that a significantly increased number of Bax-positive cells and decreased number of Bcl-2-positive cells were found in SV following cisplatin treatment [15]. Nevertheless, the underlying mechanisms of cisplatin ototoxicity are complex, and it is not clear if all the components constituting the SV (i.e., MCs, PVM/Ms, and capillary) are equally sensitive to cisplatin ototoxicity. In addition, the effect of cisplatin on cell junctions in SV, such as tight junctions (TJs) and GJs which are important in maintaining ion concentration gradient between the endolymph and the perilymph, is unknown yet.

In view of the above, we hypothesized that the ototoxic effects of cisplatin could be induced by an alteration of cell junctions of the endothelial layer. Here, the mice were injected intraperitoneally (i.p.) with 3 mg/kg cisplatin for 7 consecutive days, and we found that cisplatin damaged the SV via changing expressions of cell junction proteins zonula occludens protein-1 (ZO-1), connexin 26 (Cx26), and connexin 43 (Cx43). More importantly, we identified that cisplatin induced interleukin-1beta (IL-1*β*) activation and increased the expression of profibrotic proteins such as laminin and collagen IV, as well as led to structural morphologic alterations of the cell junction component in SV. This work establishes a basis for exploring the underlying mechanisms of cisplatin-induced SV injury and might offer novel therapeutic targets for the prevention of hearing loss resulting from SV dysfunction.

## 2. Materials and Methods

### 2.1. Animals and Cisplatin Administration

C57BL/6 mice were obtained from the Animal Center of Shandong University (Jinan, China). The mice were housed in a temperature-controlled (20-22°C) room and a 12/12 h light/dark cycle and had free access to food and drinking water. Experiments were performed on age- and sex-matched 6- to 8-week-old mice weighing 17-23 g. After acclimation for a week, the mice were randomly divided into two groups (*n* = 18 per group) depending on the administered cisplatin (Jiangsu Haosen Pharmaceutical Co. Ltd., Jiangsu, China) dose as follows: control group, the mice were intraperitoneally (i.p.) injected with 0.9% physiological saline (the vehicle of cisplatin, 0.6 ml/100 g) once a day for 7 days; cisplatin group, the mice were i.p. injected with 3 mg/kg cisplatin in 0.9% physiological saline once a day for 7 days.

All study protocols were approved by the Animal Care Committee of Shandong University and conformed with the Guideline for the Care and Use of Laboratory Animal of the National institutes of Health.

### 2.2. Auditory Function Evaluation

The auditory function of mice was evaluated by auditory brainstem response (ABR) analysis. ABR responses were measured with a tone burst stimulus at 4, 8, 12, 16, 24, and 32 kHz using the TDT system 3 (Tucker-Davis Technologies, Alachua, FL, USA) with 1024 stimulus repetitions per record in a sound isolation booth. Briefly, a total of 36 mice (*n* = 18 per group) were anesthetized with a mixture of xylazine (10 mg/kg) and ketamine (100 mg/kg) by i.p. injection and placed on a warm heating pad during ABR recordings. The sound delivery tube of an inserted earphone was tightly fitted into the external auditory canal; needle electrodes were inserted into subcutaneous tissue at the vertex (record electrode), ipsilateral ear (reference electrode), and back (ground electrode). The sound level started from a 90 dB sound pressure level (SPL) and decreased in 5 dB increments to the acoustic threshold. At each frequency, the ABR threshold was determined, which refers to the minimal SPL resulting in a reliable ABR recording with one or more distinguishable waves that can be clearly identified by visual inspection. It is necessary to repeat the process for low SPLs around the threshold to ensure the consistency of the waveforms. Following the ABR hearing measurements, tissues from the same mice were used to conduct histological, biochemical, or molecular analyses.

### 2.3. Endocochlear Potential Recording

Mice were anaesthetized as described above, and body temperature was maintained at 37°C using a heating pad. Then, the cochlea was exposed by a ventral approach and the bone over the spiral ligament of the basal turn was gently picked to form a small hole (~30 *μ*m). A glass pipette as the record electrode filled with 3 M KCl with the resistance of 12-20 M*Ω* was installed on a motorized manipulator (IVM Single, Scientifica Limited, UK). An Axopatch 200B amplifier (Molecular Devices, LLC., USA) with an Axon Digidata 1550B interfaced by software pCLAMP (version 10.6, Molecular Devices, LLC., USA) was used for the current-clamp recording of EP. When the microelectrode advanced through the round window membrane into the scala tympani, the potential was set to zero as the baseline. Then, the microelectrode was advanced into the scala media through the basilar membrane to record EP. The reference electrode was inserted in the neck muscles of the mice.

### 2.4. Sample Collection

Following the ABR measurements, the mice were sacrificed by cervical dislocation and the temporal bones were excised from the head. After removal of stapes from the oval window and piercing of the round window and the cochlear top, the cochleae were fixed in 4% paraformaldehyde (PFA) (Sigma-Aldrich, St. Louis, MO) overnight at 4°C and then rinsed in 37°C phosphate-buffered saline (PBS, pH 7.4) to remove any residual PFA. The right cochleae were decalcified in PBS containing 10% ethylenediaminetetraacetic acid (EDTA) for 30 hours at 4°C, dehydrated by successive incubation in 10%, 20%, and 30% sucrose in PBS, and embedded with O.C.T compound (Tissue-Tek, Sakura Finetek, USA) for frozen sections. Frozen sections were cut into 7 *μ*m sections using a cryostat (Leica CM1850, Leica, Nussloch, Germany). The lateral wall of the left cochlea was microdissected from the bony wall of the cochlea. Localizing the pigmented strip in the cochlear lateral wall, the SV was gently microdissected from the spiral ligament using fine forceps and adhered on a small slide precoated with celltack for immunofluorescence staining.

### 2.5. Immunofluorescence Staining

The SV tissues and frozen sections were permeabilized in 0.5% Triton X-100 (Sigma-Aldrich) for 1 hour and immunoblocked with 1% fish gelatin solution (G7765, Sigma) for another 1 hour. The specimens were incubated with different primary antibodies: ZO-1 (1 : 1000; 61-7300, Thermo Fisher), Cx26 (1 : 200; LS-B6429-50, Life Technologies), Cx43 (1 : 400; Ab135763, Abcam), laminin (1 : 200; Ab30320, Abcam), collagen IV (1 : 200; Ab6586, Abcam), alpha-smooth muscle actin (*α*-SMA, 1 : 200; Ab5694, Abcam), and CD68 (1 : 100; Ab53444, Abcam), diluted in 1% bovine serum albumin- (BSA-) PBS, respectively, at 4°C overnight. The next day, cells were incubated with secondary antibodies (Invitrogen, A21202, A21206, A31571, A31573, and A10040) along with DAPI (D9542, Sigma-Aldrich, USA) at room temperature for 1 h. Coverslips were then mounted, and the samples were observed under a laser scanning confocal microscope (Leica SP8; Leica, Germany).

### 2.6. TEM

Animals were decapitated under deep anesthesia, and the cochlear tissue was collected, washed fast with PBS, and immediately placed in 3% glutaraldehyde fixative solution (pH 7.4). The sample block was trimmed 1 mm × 1 mm × 3 mm, according to the conventional TEM sample preparation method followed by rinsing, as well as 1% osmic acid (OsO_4_) fixed, dehydrated, soaked, and epon 812 embedded; semi- and ultrathin radial sections were cut from the basal turn with lead citrate and uranyl acetate electron staining. Finally, the strial sections were observed using a transmission electron microscope (JEOL 1200EX, Japan) in Jinan WeiYa Bio-Technology Co., Ltd. (Jinan, China).

### 2.7. mRNA Extraction and Real-Time Polymerase Chain Reaction (PCR)

The total RNA of the SV was extracted with the RNA extraction kit (RNeasy Mini QIAcube Kit, Qiagen, Valencia, CA). The relative expression levels of target gene mRNA were measured by real-time PCR using an Eppendorf AG 22331 PCR machine (Hamburg, Germany). The 20 *μ*l real-time PCR reaction system included 2x SYBR Green Premix EX Taq 10 *μ*l (RR42LR, Takara Biotech), 1 *μ*g cDNA template, 1 *μ*l forward primer, and 1 *μ*l reverse primer, with deionized water complementing for the rest of the volume. The real-time PCR parameters were predegenerated at 95°C for 3 min, then 40 cycles of degeneration at 95°C for 50 s, annealing at 60°C for 45 s, and elongation at 72°C for 50 s. The specificity of each PCR reaction was confirmed by melting curve analysis. The sequences of primers used in this experiment were listed below: *zo*-*1*, forward primer: 5′ CTCCAGTCAGCCCGCAAAG 3′, reverse primer: 5′ CAAGACAACATCCCCTTCTTGA 3′; *cx26*, forward primer: 5′ TCGGGGGTGTCAACAAACAC 3′, reverse primer: 5′ CGTAGCATACATTCTTGCAGCC 3′; *cx43*, forward primer: 5′ TGGCTGTCGGTGCTCTTCATT 3′, reverse primer: 5′ GTGGGCACAGACACGAATATGAT 3′; *collagen IV*, forward primer: 5′ AATCCCAGGAGGACGAGGTGT 3′, reverse primer: 5′ GGATTACCCACTTGCCCCCAG 3′; *laminin*, forward primer: 5′ GACCTCCCACTTACAGAGCAG 3′, reverse primer: 5′ TGTACCGTGCTGAGGTGAAT 3′; *IL*-*1β*, forward primer: 5′ GATGAAGGGCTGCTTCCAAACC 3′, reverse primer: 5′ GGTGCTCATGTCCTCATCCTGG 3′; and *β*-*actin*, forward primer: 5′ GTCCCTCACCCTCCCAAAAG 3′, reverse primer: 5′GCTGCCTCAACACCTCAACCC3 ′.

### 2.8. Protein Extraction and Western Blotting

The total protein of SV was extracted with cold RIPA lysis buffer (P0013B; Beyotime Institute of Biotechnology, Shanghai, China) plus protease inhibitor cocktail (P8340; Sigma, USA) for 30 minutes at 4°C and then centrifuged at 12000 g for 20 minutes at 4°C. Protein concentrations were detected by the BCA Protein Assay Kit (Shenergy Biocolor Bioscience & Technology Company, Shanghai, China). The same amount of protein samples was denatured in 99°C for 10 min and separated by 10% SDS-PAGE gel electrophoresis. Then, the proteins were transferred to polyvinylidene difluoride membranes. The membranes were blocked in 5% skim milk for 1 hour at room temperature and then were incubated with different primary antibodies: ZO-1 (1 : 200; 61-7300, Thermo Fisher), connexin 26 (Cx26, 1 : 200; LS-B6429-50, Life Technologies), connexin 43 (Cx43, 1 : 200; Ab135763), laminin (1 : 200; Ab30320, Abcam), collagen IV (1 : 200; Ab6586, Abcam), and anti-*β*-actin antibody (1 : 2000 dilution; ZSGB-BIO, Beijing, China) in 3% BSA at 4°C overnight. The next day, the membranes were incubated with HRP-conjugated goat anti-mouse or goat anti-rabbit IgG antibodies (1 : 2000 dilution; ZSGB-BIO, Beijing, China) at room temperature for 1 hour. Finally, the protein signals were detected using an ECL kit (Millipore, Billerica, MA, USA) and analyzed by ImageJ software. All blocking, incubation, and washing were performed in TBST solution (Tris-buffered saline and 0.05% Tween 20).

### 2.9. Cell Counting

As for cell quantification, we imaged the entire cochlea using a 400 × 2.5 objective and used ImageJ software to quantify the immunostaining-positive cells. In detail, we measured the relative length of the scale bar (20 *μ*m) in the figure and set scale to 20 *μ*m in the ImageJ software. Then, we used the “point selections” tool in the ImageJ to count the immunostaining-positive cell number.

### 2.10. Statistical Analysis

All statistical analysis was performed with SPSS 13.0 software (SPSS Inc., USA). Results were expressed as mean ± standard deviation (SD). Statistical comparisons were performed by independent-samples *t*-tests. Differences with a *p* value < 0.05 were considered to be statistically significant.

## 3. Results

### 3.1. Cisplatin Induces Insult to Auditory Function

**Figure 1:**
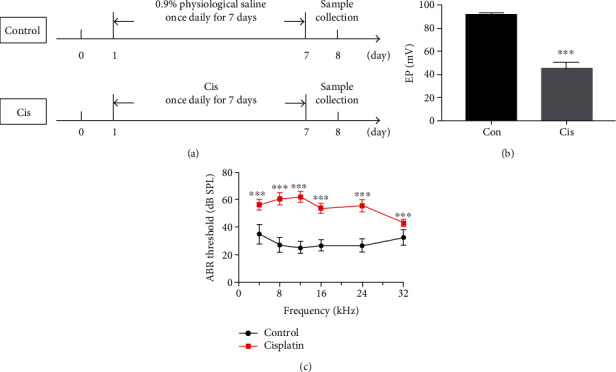
Cisplatin induced insult to auditory function. (a) C57BL/6 mice were divided into 2 groups and given i.p. injection of drugs as illustrated. (b) Endocochlear potentials were measured 7 days after cisplatin administration (3 mg/day/kg, i.p.). The EP in cisplatin-treated mice was significantly reduced compared to the control group (*n* = 3). (c) ABR thresholds were measured at 4, 8, 12, 16, 24, and 48 kHz in the control and cisplatin groups, and the ABR thresholds were elevated in the cisplatin group compared to the control group at all frequencies measured (*n* = 18). Data are shown as mean ± SD. Cis: cisplatin; ^∗∗∗^*p* < 0.001.

To investigate the effect of cisplatin on auditory function, C57BL/6 mice at 6-8 weeks were given i.p. injections of cisplatin for 7 days (Figure 1(a)), and we found that the EP in cisplatin-treated mice was significantly reduced compared to the control group (Figure 1(b), *p* < 0.001), as EP in the control and cisplatin-treated mice was 91.3 ± 1.53 mV and 44.8 ± 5.62 mV, respectively. The auditory functions of mice were evaluated using the ABR test. The ABR thresholds were elevated in the cisplatin group compared to the control group at all frequencies measured (Figure 1(c), *p* < 0.001), suggesting that cisplatin effectively impaired auditory function in mice in our study.

### 3.2. Cisplatin Changes the Ultrastructure of SV Cells

**Figure 2:**
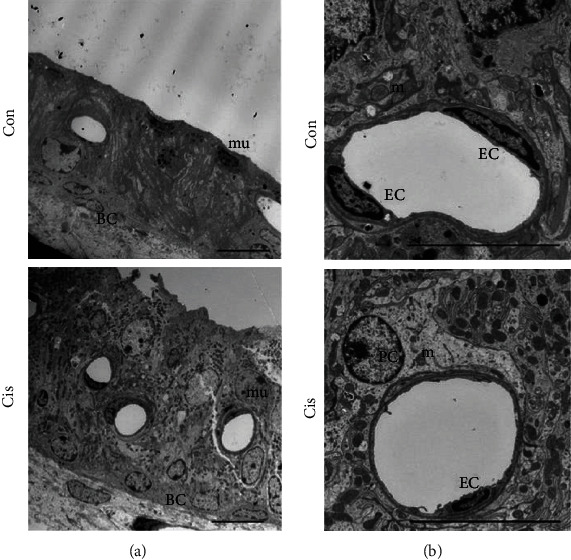
Cisplatin changes the ultrastructure of SV cells. (a, b) The ultrastructure of SV was observed by TEM. Both the size and the shape of cell nuclei appeared normal, and mitochondria were prevalent and occurred in a highly organized manner in the control mice. A thicker SV and more blood vessels were observed in the cisplatin-treated mice; the numbers of mitochondria and endothelial cells were reduced; and most of the cells were in an abnormal shapes with large and round nuclei, a swollen endoplasmic reticulum, and a thinner vascular wall in cisplatin-treated mice. Con: 0.9% physiological saline; Cis: cisplatin; EC: endothelial cell; m: mitochondrion; PC: pericyte; mu: marginal cell nucleus. Scale bars, 10 *μ*m.

The ultrastructure of SV was observed by TEM after cisplatin administration. In the control tissues, the SV was in a normal morphology, as indicated by the characteristic differences in electron density between the darker MCs with their finger-like processes and the lighter PVM/Ms and BCs (Figure 2(a)). Both the size and shape of cell nuclei appeared normal, with nucleoli and proportional amounts of euchromatin and denser heterochromatin (Figure 2(a)). However, a thicker SV and more blood vessels were observed in the cisplatin-treated mice compared to the control group. It should be noted here that most of the nuclei, which were very light, were abnormal in shape. Especially in the MCs, the nuclei were large and round rather than oval and often with indistinguishable and disorganized nucleolus; moreover, the MCs were atrophied and the cell-cell connections were interrupted (Figure 2(a)). Moreover, our analyses revealed that the number of mitochondria was much lower in SV of cisplatin-treated mice compared with the control ones, in which mitochondria were prevalent and occurred in a highly organized manner (Figure 2(a)). Besides, analysis of ultrathin sections showed that cisplatin treatment led to reduced endothelial cells, a swollen endoplasmic reticulum, and a thinner vascular wall compared to control mice (Figure 2(b)). We also observed changes in pericytes migrating away from blood vessels (Figure 2(b)).

### 3.3. Cisplatin Alters the Expression Pattern and Decreases the Expression of ZO-1 in SV

**Figure 3:**
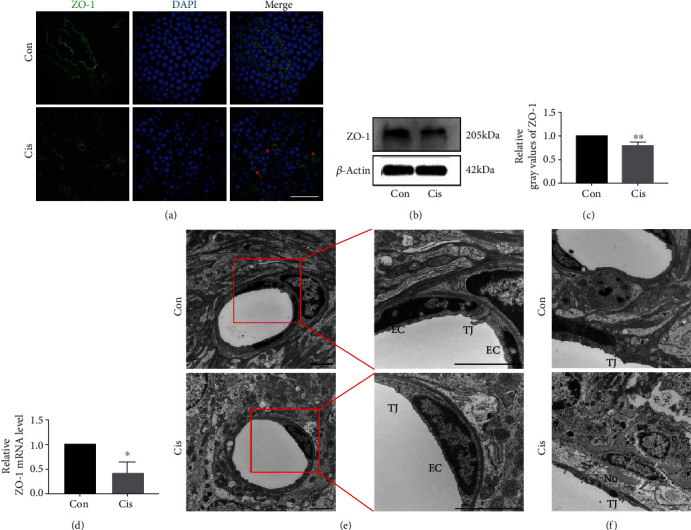
Cisplatin alters the expression pattern and decreases the expression of ZO-1 in SV. (a) The distribution of ZO-1 (green) in MCs by immunofluorescence staining. The moderate-to-strong ZO-1 signal is visible as a continuous wavy line between adjacent MCs in control mice, while the weak-to-moderate ZO-1 signal is seen mainly in the cell cytoplasm in cisplatin-treated mice. Asterisks show holes formed between marginal cells. Arrows point to morphological alterations in intercellular junctions. Nuclei were stained with DAPI (blue). Scale bars, 100 *μ*m. (b) Western blot analysis of the cell lysates of SV treated with cisplatin. *β*-Actin was used as a loading control. (c) Densitometric quantification of the mean (SD) ratio of ZO-1 expressed as mean ± SD (*n* = 3). (d) qRT-PCR shows mRNA for ZO-1 in the SV (*n* = 3). (e) The ultrastructure of SV was analyzed by TEM. TJs between adjacent ECs were extremely tight in control mice; the right window (zoomed inset) displays a higher magnification of the TJs between ECs; TJs were open and intermittently widened between adjacent ECs in cisplatin-treated mice. Scale bars, 2 *μ*m. (f) TJs between adjacent MCs were extremely tight in the control mice; TJs were open between adjacent MCs in cisplatin-treated mice. Scale bars, 2 *μ*m. Con: 0.9% physiological saline; Cis: cisplatin; TJ: tight junction; EC: endothelial cell; m: mitochondrion; Nu: marginal cell nucleus. ^∗^*p* < 0.05, ^∗∗^*p* < 0.01, and ^∗∗∗^*p* < 0.001.

Immunofluorescence staining was used to assess the effect of cisplatin on the typical localization pattern of tight junction protein ZO-1 after 7 days of administration. In control mice, the ZO-1 signal appeared at the cell surface and localized to the cell-cell junction of MCs, with a more prominent and clear immunostaining at the intercellular border (Figure 3(a)), which clearly suggested the presence of physiological tightness of the barrier. However, the exposure of MCs to cisplatin resulted in protein delocalization, leading to a “zipper-like” staining pattern and holes that became visible between cells (Figure 3(a)). In addition, western blotting and qPCR results revealed significant decreases of ZO-1 expression in cisplatin-treated mice compared to the control group (Figures 3(b)–3(d)), indicating that the TJ between MCs was damaged after cisplatin treatment.

Furthermore, the TEM analyses also showed that the number of cell projections interdigitated and TJs coexisting with the MCs or located in their vicinity were distinctly reduced in cisplatin-treated mice compared with the controls (Figure 3(e)). In the classic view, the intrastrial fluid-blood barrier is composed of the basement membrane and endothelial cells that connect to each other with TJs [16] to form a diffusion barrier that prevents most blood-borne substances from entering the ear [17]. Observations revealed the presence of endothelial cell nuclei with irregular morphology, intact organelles in the cytoplasm, and intact TJ structure between endothelial cells (Figures 3(e) and 3(f)). After cisplatin exposure for 7 days, the TJs were open and intermittently widened between adjacent ECs (Figures 3(e) and 3(f)), indicating that the cisplatin ototoxicity was associated with an altered TJ organization.

### 3.4. Cisplatin Reduces Expressions of Cx26 and Cx43 in SV

**Figure 4:**
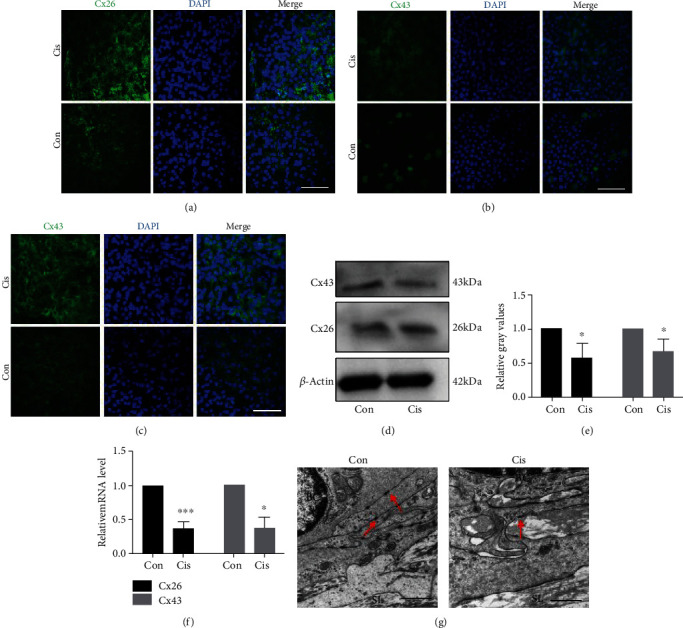
Cisplatin reduces expressions of Cx26 and Cx43 in SV. (a) The distribution of Cx26 (green) in BCs by immunofluorescence staining. The moderate-to-strong Cx26 signal is visible in the cell cytoplasm of BCs in the control, while the weak-to-moderate Cx26 signal is seen in cisplatin-treated mice. Nuclei were stained with DAPI (blue). Scale bars, 100 *μ*m. (b, c). The distribution of Cx43 (green) in the MCs (b) and BCs (c) by immunofluorescence staining. After the treatment of cisplatin, the weak-to-moderate Cx43 signal was found in the SV. A strong signal for Cx43 was found in some MCs in cisplatin-treated mice. Nuclei were stained with DAPI (blue). Scale bars, 100 *μ*m. (d) Western blot analysis of the cell lysates of SV treated with cisplatin. *β*-Actin was used as a loading control. (e) Densitometric quantification of the mean (SD) ratio of Cx43 and Cx26 expressed as mean ± SD (*n* = 3). (f) qRT-PCR shows mRNA for Cx43 and Cx26 in the SV (*n* = 3). (g) The ultrastructure of SV analyzed by TEM. The number of anchoring connections at the basal layer is reduced in cisplatin-treated mice. Arrows point to anchoring connections. Scale bars, 10 *μ*m. BC: basal cells; SL: spiral ligament; Con: 0.9% physiological saline; Cis: cisplatin. ^∗^*p* < 0.05, ^∗∗^*p* < 0.01, and ^∗∗∗^*p* < 0.001.

To assess the effect of cisplatin on GJ protein expression, immunofluorescence staining and TEM were used after the mice were treated with cisplatin injection. As revealed by immunofluorescence, Cx26 was mainly flocculently expressed in the basal layer and Cx43 was expressed in the MCs and BCs, and they were both localized in the cytoplasm of cells (Figures 4(a)–4(c)). Cisplatin treatment resulted in frequently dispersed staining of Cx26 in the cell cytoplasm of BCs (Figure 4(a)), as well as the reduction of Cx43 signal intensity (Figures 4(b) and 4(c)). Within some MCs, however, a very strong linear Cx43 signal on the plasma membrane of MCs was found as the prevalent staining pattern compared with the control (Figure 4(b)). Further quantitative evaluation of the Cx26 and Cx43 protein and mRNA expression by western blotting and qPCR also reflected the qualitative results (Figures 4(d)–4(f)).

The results of TEM showed that the number of anchoring connections at the basal layer was reduced (Figure 4(g)), and the number of GJs coexisting with the BCs or located in their vicinity was reduced in cisplatin-treated mice compared with the controls. Similar reduction was also observed in the case of the spiral ligament that, as a rule, contained only single or few TJs (Figure 4(g)). After cisplatin treatment, the expression and number of GJs were reduced significantly, indicating that the GJs were damaged in SV.

### 3.5. Cisplatin Promotes Translocalization of *α*-SMA-Positive Cells into SV and Increases the Expression of Collagen IV and Laminin

**Figure 5:**
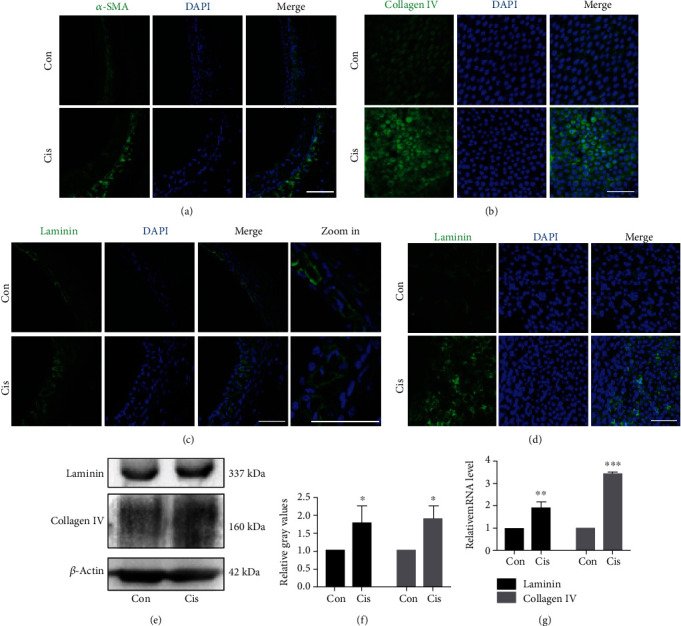
Cisplatin promotes translocalization of *α*-SMA-positive cells into SV and increases the expression of collagen IV and laminin. (a) Confocal immunofluorescence for *α*-SMA in lateral tissue. Nuclei were stained with DAPI (blue). Scale bars, 100 *μ*m. (b) The distribution of collagen IV (green) in the MCs by immunofluorescence staining. After the treatment of cisplatin, the weak-to-moderate collagen IV signal was found in the SV. Nuclei were stained with DAPI (blue). Scale bars, 100 *μ*m. (c, d) The distribution of laminin (green) in frozen sections (c) and surface preparation (d) by immunofluorescence staining. After the treatment of cisplatin, the expression of laminin increased after cisplatin treatment, blood vessel cavity became rounder, and pericytes migrated away from blood vessels. Scale bars, 100 *μ*m. (e) Western blot of cell lysates of SV treated with cisplatin. *β*-Actin was used as a loading control. (g) qRT-PCR shows mRNA for laminin and collagen IV in the SV (*n* = 3). Con: 0.9% physiological saline; Cis: cisplatin. ^∗^*p* < 0.05, ^∗∗^*p* < 0.01, and ^∗∗∗^*p* < 0.001.

Myofibroblasts are a subset of activated fibroblasts, characterized by expression of *α*-SMA, which is the principal cell type responsible for excessive extracellular matrix (ECM) deposition [18]. We found that cisplatin increased the translocalization of *α*-SMA-positive cells into SV compared to the control mice (Figure 5(a)). These data suggested that cisplatin exerted a promotive effect on transformation of fibroblasts into myofibroblasts during the fibrogenesis process.

We next investigated whether markers of ECM were increased in SV after cisplatin administration so as to determine the effects of cisplatin on fibrogenesis in mice. Collagen IV was mainly expressed in the cytoplasm of MCs (Figure 5(b)), and the increased protein and mRNA levels of collagen IV were found remarkably after cisplatin treatment (Figures 5(e)–5(g)). We also observed a significant increased expression of laminin (Figures 5(d)–5(g)), an analyzed marker of vascular lesions. In accordance with the TEM result in Figure 2(d), the blood vessel cavity became rounder, and pericytes migrated away from blood vessels (Figure 5(c)). In brief, our data indicated that fibrosis in SV was induced after cisplatin treatment.

### 3.6. Cisplatin Activates PVM/Ms in SV

**Figure 6:**
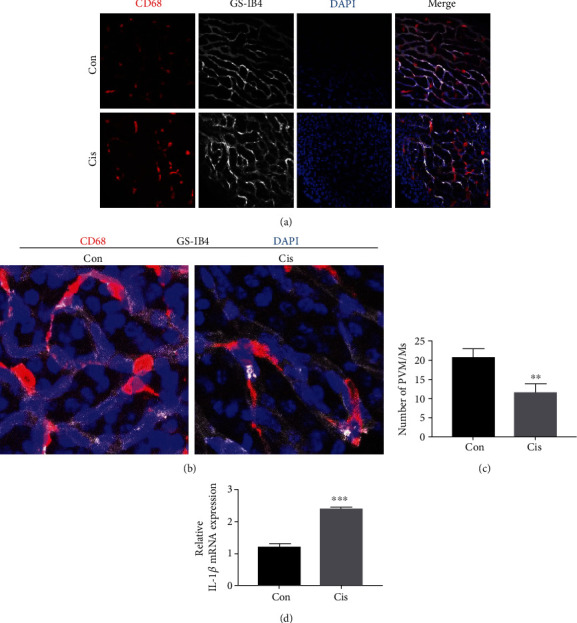
Cisplatin activates PVM/Ms in SV. (a) The distribution of PVM/Ms by immunofluorescence staining. PVM/Ms were marked with a special antibody against CD68 (red) and strial capillaries with Griffonia Simplicifolia IB4 (gray). Nuclei were stained with DAPI (blue). The PVM/Ms were highly invested on the abluminal surface of capillaries. Scale bars, 100 *μ*m. (b) Zoom in of immunofluorescence staining. (c) Quantification of CD68-positive infiltration per SV area (*n* = 5). (d) qRT-PCR shows mRNA for IL-1*β* in the SV. *n* = 6. ^∗∗^*p* < 0.01 and ^∗∗∗^*p* < 0.001.

Studies have shown that PVM/Ms control barrier integrity by affecting the expression of tight junction-associated proteins [19]. PVM/Ms were marked with a special antibody against CD68 and strial capillaries with Griffonia Simplicifolia IB4 (GS-IB4; Figure 6(a)). We found that the PVM/Ms were highly invested on the abluminal surface of capillaries (Figure 6(a)). The number of PVM/Ms in the SV decreased statistically after cisplatin treatment in mice compared with the control group (Figure 6(c)). Immunofluorescence results showed that the morphology of PVM/Ms was changed, the volume of PVM/Ms was enlarged, and the number of PVM/Ms branches was increased, as well as some of the branches became flat and amoeboid shaped (Figure 6(b)). The relative mRNA level of proinflammatory cytokine IL-1*β* secreted by PVM/Ms was also significantly increased (Figure 6(d)). Together, these data suggested that PVM/Ms and their secreted IL-1*β* may be involved in the damage of cell junction in SV induced by cisplatin.

## 4. Discussion

Hearing loss is one of the most common sensory disorder in human population, which affects 466 million people all over the world. Hearing loss can be caused by a variety of different stresses and injuries, such as genetic disorders, aging, noise acoustic trauma, inflammation, infection, and exposure to different ototoxic drugs, including cisplatin [20–28]. Unfortunately, once the inner ear cochlea was damaged, the mammals only have very limited regeneration ability, which makes hearing loss irreversible in mammals [29–35]. Hearing loss significantly decreases the patient's life quality; and for the children, hearing loss will delay the language acquisition and affect the learning ability. Thus, to protect the cochlea and the hearing ability is very important.

Cisplatin-induced reduction of EP, which is generated and maintained by SV processes, has been observed after application of single high-dose cisplatin injection [36], following repeated systemic administration [37] and during perilymphatic administration [38]. In our previous study, we observed that 3 mg/kg/day i.p. injection of cisplatin for 7 consecutive days could cause obvious damages to the spiral ganglion neurons, vestibular HCs, and impairments to the cochlea [11, 39, 40]. Although studies in rodents have established cisplatin-dependent inner ear dysfunction and there are now evidences suggesting that cisplatin exerts cytotoxic effect on the SV, whether cisplatin alters the cell-cell junctions in the SV remains elusive. In this study, for the first time, we found that the treatment of cisplatin caused SV lesions in mice, which suggested that the cisplatin-induced SV damage might be associated with inflammation and the fibrosis process.

In general, cisplatin depletes cytoplasmic organelles, damages the mitochondria, and causes formation of lipid bodies and vacuoles [41]. The mainly impacted targets of cisplatin in SV are the MCs and the ICs [42, 43]. In our study, we found that the EP in cisplatin-treated mice was significantly reduced compared to that in the control group, suggesting that cisplatin damaged the SV and thus impaired the auditory function in mice. Consistently, our TEM results showed that the subnuclear processes, endoplasmic reticulum, nuclei, and mitochondria of the SV were extensively damaged by cisplatin. It has been reported that after 30 min i.p. infusion of 16 mg/kg cisplatin, SV edema can be present [44]. We found that cisplatin treatment (3 mg/kg/day for 7 days) causes SV edema in basal regions. Unlike the extracellular edema that is generally observed secondary to loop diuretics [45], the edema secondary to cisplatin appears to be intracellular which could account for the bulging and rupturing of MCs [42]. Strial capillaries have a minor role in blood flow regulation but a crucial role in maintaining the EP, ion transport, and endolymphatic fluid balance [1, 2]. We found that cisplatin treatment led to a reduced number of endothelial cells and thinner vascular wall. Three-dimensional FLAIR imaging detected that the blood-labyrinth barrier (BLB) breaks down with the enhancement of the endolabyrinthic fluid signal in patients with sudden hearing loss [46]. Thus, the edema of SV induced by cisplatin might also result in BLB breakdown and increase vascular permeability. Moreover, we found an increase in vessel density after cisplatin treatment. A study has reported that in temporal lobe epilepsy patients, the vessel density was associated with hypoxia and inflammation [47]. But there has been hardly any evidence on the vascular remodeling affected by cisplatin, and the role of angiogenesis is instead unclear, which needs to be further studied.

TJs are located on the lateral side of epithelial cells, forming the structural basis of selective cellular permeability. Like in other barrier systems, the intrastrial fluid-blood barrier is a highly specialized vascular epithelium composed of the basement membrane and ECs formed a diffusion barrier that prevents transport of most blood-borne substances into the inner ear [17]. TJs in the SV are also important in maintaining the ion concentration gradient between the endolymph and the perilymph [5]. ZO-1 is a recognition protein for TJ placement, and loss of ZO-1 results in disorganization of TJs [48]. In this study, we found that the ZO-1 signal appeared at the cell surfaces and localized to the cell-cell junction of MCs, which suggested the presence of the physiological tightness of the barrier. After cisplatin exposure, ZO-1 expression was reduced with impaired functionality of SV. This idea is strengthened by the TEM results. Therefore, these results suggest that cisplatin could destroy the TJ organization between MCs and vascular endothelial cells and increase the permeability of the SV.

GJ proteins are specific connexins in the membrane of neighboring cells, responsible for exchanging of electric and chemical signals between cells. Cx26 is seen in GJs of all cells in the cochlea which participates in the circulation of potassium ions from HCs to MCs of the SV and the maintenance of cellular homeostasis [49]. A major portion (>50%) of nonsyndromic hereditary deafness is caused by Cx26 gene mutations. Loss of Cx26 results in congenital hearing loss and cochlear development failure, as well as dysplasia of HCs [50]. *Cx43* gene is expressed in the mouse inner ear and plays a role in the vascular system [51]. In our study, cisplatin treatment resulted in frequently dispersed staining of Cx26 and Cx43 in the cell cytoplasm. The number of anchoring connections at the basal layer of SV and spiral ligament was reduced, indicating that the GJs were damaged in the SV and spiral ligament. We suggest that the reduction of the cell junction protein is a consequence of internalization into the cytoplasm. Indeed, the reduction of the ZO-1 signal and its translocalization from the membrane to the cytoplasmic compartment was reported to be physically associated with degradation of the GJ organization in the Sertoli cell line [3]. Thus, the diminished expression of Cx43 of cisplatin-treated mice may result, at least in part, from the decreased ZO-1 expression. Our results allow us to propose a working model: the cell junction of SV is damaged by cisplatin, thus leading to an increased permeability, allowing uncontrolled passage of ions and proteins, which together allow more cisplatin to enter into the inner ear and, in turn, provoke severe ototoxicity and impairment in auditory function.

It was important to give an answer whether and how the expression of TJs is related to the changes of the GJ expression observed in cisplatin-treated mice, and the question aroused whether diminished TJ and GJ protein expression in SV may be related to structural changes seen at the ultrastructural level, which are currently under study in this laboratory.

PVM/Ms are regulatory cells in the intrastrial fluid-blood barrier which are in close contact with vessels and critical for barrier function. Studies have shown that PVM/Ms affect barrier integrity by affecting the expression of TJ-associated proteins, and loss of PVM/Ms is associated with tissue edema [19]. In this study, we observed that the number of CD68-positive cells was decreased in SV after cisplatin treatment, but the remaining cells became bigger and longer in size, suggesting cisplatin could activate and damage PVM/Ms at the same time. Some studies suggested that cisplatin promoted excessive production of reactive oxygen species and proapoptotic factors in cochlear cells, leading to cell apoptosis through caspase activation [11, 15, 52]. We speculate that cisplatin-triggered activation of PVM/Ms, by its effect on extending cytokine production, could also extend cisplatin-induced inner ear inflammation. The specific signals underlying PVM/M activation are still not fully clear. IL-1*β* is a major proinflammatory cytokine which is produced by activated macrophages and is widely regarded as a hallmark of inflammatory gene cascades [53]. A recent study also revealed that PVM/Ms in the mouse cochlea could activate the NLRP3 inflammasome in response to stimulation with lipopolysaccharide plus adenosine triphosphate, which results in IL-1*β* secretion [54]. We found that the IL-1*β* was also significantly increased after cisplatin treatment in SV. In our preliminary experiments, cytokines such as IL-2, IL-4, and IL-10 in SV were also detected in mice after cisplatin treatment; however, the results showed that there were no significant differences of these cytokines in SV between the control group and the cisplatin-treated group (data not shown). We speculate that excessive expression and/or aberrant activation of IL-1*β* and PVM/Ms in the cochlea might contribute to the deterioration of hearing function, which needs to be investigated in further experiments.

Increased levels of laminin immunoreactivity have been reported in blood vessel basal lamina, following cerebral ischemia [55] and quinolinic acid [56] treatments. Notably, a similar upregulation in laminin and collagen IV levels is found in mice injected with cisplatin. The laminin overexpression is explained by an improved accessibility of laminin epitope disconnection of the gliovascular junctions in damaged blood vessels [57]. According to these authors, gliovascular junctions could limit the accessibility of the basal lamina of blood vessels to antibodies, thus “hiding” the laminin epitopes. Therefore, upregulation of laminin and collagen IV expression is one of the consequences from BLB modification and cisplatin promotes the progression of SV fibrosis by increasing the expression of ECM components. The presence of *α*-SMA-positive myofibroblast-like cells is considered a hallmark for the development of kidney fibrosis [18]. In our study, we found increased localization of *α*-SMA-positive cells in the SV. These results are in agreement with *in vitro* and *in vivo* studies that have shown areas of fibrosis-activated macrophages generating soluble mediators that modulate activation and proliferation of myofibroblasts [58, 59]. Therefore, our findings suggest that cisplatin might promote the progression of SV fibrosis through the regulation of macrophage infiltration, secretion of IL-1*β*, and accumulation of laminin and collagen IV.

In summary, the present investigation showed that there were structural morphologic alterations of the cell junction component in the mice SV; TJ and GJ proteins were downregulated substantially after cisplatin treatment. Our findings suggested that the changes induced by cisplatin may be associated with PVM/Ms activation, IL-1*β* secretion, and ECM overexpression. The results reported herein provide evidence that the interaction of cisplatin with the BLB in SV results in a translocation of the cell junction apparatus that, in turn, could result in secondary injuries to the inner ear. Further investigation on the molecular mechanism underlying cisplatin-induced SV damage could bring new targets to develop specific therapies against cisplatin ototoxicity.

## Data Availability

The datasets used and analyzed during the current study are available from the corresponding authors on reasonable request.
